# Perspectives on the Current and Future State of Artificial Intelligence in Medical Genetics

**DOI:** 10.1002/ajmg.a.64118

**Published:** 2025-05-15

**Authors:** Benjamin D. Solomon, Morgan Cheatham, Thales A. C. de Guimarães, Dat Duong, Melissa A. Haendel, Tzung-Chien Hsieh, Behnam Javanmardi, Britt Johnson, Peter Krawitz, Paul Kruszka, Tim Laurent, Ni-Chung Lee, Kirsty McWalter, Michel Michaelides, Klaus Mohnike, Nikolas Pontikos, Maria J. Guillen Sacoto, Yousif J. Shwetar, Vincent D. Ustach, Rebekah L. Waikel, William Woof

**Affiliations:** 1 Medical Genomics Unit, National Human Genome Research Institute, Bethesda, Maryland, USA; 2 Warren Alpert Medical School of Brown University, Providence, Rhode Island, USA; 3 Moorfields Eye Hospital National Health Service Foundation Trust, London, UK; 4 University College London Institute of Ophthalmology, London, UK; 5 National Institute for Health and Care Research Moorfields Biomedical Research Centre, London, UK; 6 Department of Genetics, University of North Carolina at Chapel Hill, Chapel Hill, North Carolina, USA; 7 Institute for Genomic Statistics and Bioinformatics, University Hospital Bonn, Rheinische Friedrich-Wilhelms-Universität Bonn, Bonn, Germany; 8 GeneDx, LLC, Gaithersburg, Maryland, USA; 9 Department of Pediatrics and Medical Genetics, National Taiwan University Hospital, Taipei, Taiwan; 10 Children’s Hospital, Otto-von-Guericke-University, Magdeburg, Germany

**Keywords:** AI, artificial intelligence, clinical genetics, medical genetics, medical genomics

## Abstract

Artificial intelligence (AI) is rapidly transforming numerous aspects of daily life, including clinical practice and biomedical research. In light of this rapid transformation, and in the context of medical genetics, we assembled a group of leaders in the field to respond to the question about how AI is affecting, and especially how AI will affect, medical genetics. The authors who contributed to this collection of essays intentionally represent different areas of expertise, career stages, and geographies, and include diverse types of clinicians, computer scientists, and researchers. The individual pieces cover a wide range of areas related to medical genetics; we expect that these pieces may provide helpful windows into the ways in which AI is being actively studied, used, and considered in medical genetics.

## 1 | Introduction

Artificial intelligence (AI) is being rapidly—though unevenly—integrated into innumerable aspects of daily life, including medicine and biomedical research. AI will have increasingly significant effects across virtually everything that intersects with medical genetics. An important question is thus not so much whether AI will have a profound effect, but what the effects will be, and at what pace change will occur.

With this prediction and these unknowns in mind, and reminiscent of similar endeavors (Solomon et al. 2023), we assembled a diverse group of leaders in the field to respond to the question about how AI is affecting, and especially how AI will affect, the field of medical genetics. This manuscript is not intended to serve as a standard review article on the topic of AI in medical genetics; rather, the pieces these leaders wrote are meant to represent current and medium-term predictions about AI in medical genetics; after that, our crystal balls tend to get especially tricky to decipher. These leaders represent different areas of expertise and different geographies, and the group includes clinicians (again of different types, such as those who work in clinics as well as those primarily based in clinical laboratories), computer scientists, and other types of researchers. We also intentionally assembled a group of experts who represent diverse career stages, ranging from those starting their careers to those leading relatively large groups. As with many breakthroughs, those at early career stages tend to embrace and fluently use new technologies first and are often instrumental in educating the older generations.

We left the topics and formats of the individual pieces relatively open-ended, as we wanted authors to address the overall topic as narrowly or broadly as they saw fit. Along these lines, our authors answered, unsurprisingly, in diverse ways and from different perspectives, including how AI will affect the clinic, the lab, and both general and specific areas of research.

We do not expect readers to agree with everything here—in fact, our group of authors will surely (hopefully?) not agree with each other about everything. Similarly, some pieces may naturally resonate more with certain types of readers than others, which is also a good thing—we expect that crosstalk among those who work at the bedside, at the bench, behind the computer, or elsewhere can help lead to common languages and ideas, even if hearty and healthy disagreement remains. Overall, we also hope that this collection of essays can provide interesting food for thought—and perhaps even some excitement and inspiration—for all of us in the field of medical genetics.

Before turning to the individual essays, we provide a brief introduction to key areas and concepts related to AI, which may be helpful to those less familiar with the topic. For those interested in learning more, we point readers to more comprehensive reviews on this topic (Duong and Solomon 2025; Ledgister Hanchard et al. 2022).

Overall, AI is a very broad term meant to encompass the idea of an artificial, non-organic system (typically, some kind of computer) that is able to perform actions that are impressively clever. There are many methods that may be involved in a particular AI system, and different AI systems may be used to perform many different tasks, such as playing tic-tac-toe or chess, responding to questions, suggesting products to buy, or steering a vehicle. In medical genetics, common AI tasks may include evaluating genetic variants, analyzing data such as patient photos or X-rays to help provide suggestions regarding the differential diagnosis, writing clinical reports, or looking up medical information for clinicians or patients (Duong and Solomon 2025). However, in addition to the above-mentioned examples, it is important to note that AI is already embedded in a growing number of daily activities that are often overlooked. These activities include the smartphone-based cameras that clinicians might use to take pictures of patients, the “everyday” email systems that allow clinicians to communicate with patients, the streaming music services a research intern might listen to while working on a genetics project, or the navigation system that helps a new medical genetics resident get directions to a hospital where they are rotating.

**FIGURE 1 |:**
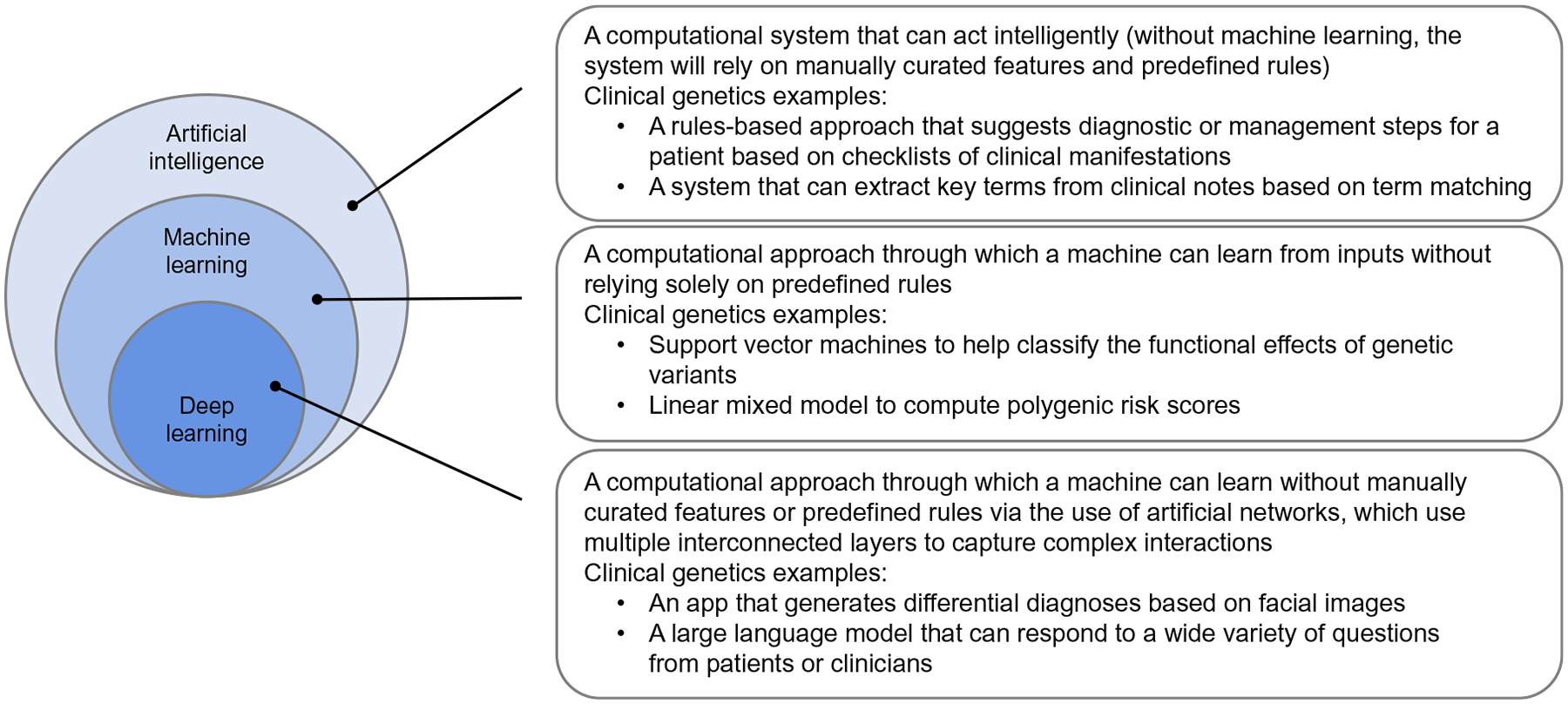
General depiction of the way that categories of artificial intelligence (AI) relate to each other, with examples of AI in medical genetics. In addition to these categories, “generative AI” includes the use of AI for the purposes of generation, such as making images, videos, or text. Image from (Duong and Solomon 2025), used with permission.

AI is not a new field, though it has gained great attention recently. The methods behind AI have evolved tremendously since the term was coined at a summer conference in the 1950s. Initial AI applications focused on using rules-based systems. Later, a type of AI called “machine learning” (ML) provided different ways for computers to learn tasks based on different types of datasets. More recently, a subtype of ML called deep learning (DL) has thrust AI in general into the limelight. In very simple terms, DL refers to a type of ML that involves the use of multiple, connected layers (hence the term deep)—the idea behind these connections is inspired by the way that the neurons in the brain connect, giving rise to the term “neural network”. DL has enabled some of the powerful—and at times controversial—AI systems that are currently upending medicine, such as DL classifiers that are very good at tasks like reading X-rays or pathology images, or large language models (LLMs) and other generative AI methods that can write clinical notes or respond to questions about pertinent topics. These generative methods, as the name implies, can be used to “make things”, whether those things are novel molecular structures that could be used to treat diseases, or written or verbal instructions for a patient’s family about how to administer medications or get access to genetic testing (Duong and Solomon 2025). See Figure 1 for brief explanations and examples about different types of AI. Beyond the very succinct descriptions here, in the pieces below, more background on certain aspects of AI will be provided in the context of the individual essay.

In the collection of essays, Cheatham first provides a broad overview of AI’s roles in diagnostics and therapeutics, including the point that AI will enable improved leveraging of multi-omic approaches in the future. As a fascinating example of how clinical data can be used alongside ‘omic information, Hsieh describes specifics of “next-generation phenotyping” (NGP), and how AI-based facial imaging and related methods can be integrated into current technologies like genomic sequencing. Hsieh discusses how NGP may be valuable in the assessment of individuals with suspected genetic conditions, but also delves into associated pitfalls. Following this specific example, Laurent et al. who represent a large clinical sequencing entity (with a substantial research footprint), provide insights into ways in which AI is used in the genomics laboratory, including for both direct genomic analyses, as well as to bolster necessary administrative and support tasks.

While there is much excitement about the potential of using AI in the ways that the first three essays describe, one fundamental challenge involves the lack of genetics experts in the United States and throughout the world. This has self-reinforcing cyclic effects: the lack of role models in genetics leads to less genetics education and exposure, which leads to a smaller workforce due to lack of role models. In light of these challenges, Waikel turns to ways in which AI can be used in medical genetics and genomics education, which may be an important way to supplement the unfortunate dearth of medical geneticists, as well as a means enable new ways to reach trainees and clinicians through alternative learning methods. Waikel includes new data that demonstrate the appetite as well as the need for AI education in genetics. Next, Haendel and Shwetar turn to the fundamental challenge of defining genetic diseases, and how painstaking work by domain experts as well as technologists are needed to allow AI systems to make sensible use of the vast troves of relevant data in electronic health record systems and related databases. Duong tackles a specific facet of AI as applied to medical genetics: the fact that there are many different genetic conditions, the vast majority of which are rare. This results in datasets that tend to be small and heterogeneous. Duong addresses recent and future methods to address these challenges so that AI can be applied effectively to the field of medical genetics.

After these relatively broad pieces, we have included several pieces that provide more glimpses into more specific aspects of AI in medical genetics. Javanmardi and colleagues describe the use of AI in genetic bone diseases, and de Guimarães and colleagues discuss AI in inherited retinal diseases. These two pieces raise several interesting questions, including how AI approaches can be tailored for subsets of genetic conditions, as well as whether and how these different AI applications might be harmonized across different areas of medical genetics. Along a similar theme of delving more deeply into specific topics, Lee provides a window onto the use and study of AI in medical genetics in Taiwan. Currently, Taiwan is often in the news due to its unique place in the AI ecosystem; it is especially illustrative to consider an example of AI in medical genetics outside of the United States and Europe.

Finally, while the above pieces involve both clinical and research-based considerations of AI, in the last perspective, Solomon focuses more squarely on the latter sphere, including predictions regarding how the discoveries that inform clinical practice may be made in a future more permeated by AI.

## 2 | Multi-Modal AI in Medical Genetics: Advancing Clinical Diagnostics and Targeted Therapeutics

### Morgan Cheatham

Medical genetics has been at the forefront of computational innovation for decades, evolving from pattern recognition algorithms for karyotype analysis to deep learning systems for variant interpretation (Chen et al. 2023; De Paoli et al. 2024; Wu et al. 1987). This evolution is not merely a product of technological opportunism, but a fundamental necessity: the complexity of genomic data and genotype–phenotype relationships demands computational approaches capable of detecting patterns in dynamic biological systems. AI will become ubiquitous in genetic medicine over the next two decades, transforming the field from primarily diagnostic to increasingly interventional, driven by exponential growth in molecular testing, availability of multi-omic data, and broader clinical and therapeutic actionability (Anderson et al. 2021; Huang et al. 2024; Sevilla et al. 2022). This transformation will inspire “genomic learning health systems” in which each patient interaction refines our genomic understanding and informs diagnostic, therapeutic, and clinical workflows (Friedman et al. 2015; Green et al. 2020).

### Multi-Modal AI Systems: Redefining Genetic Disease Models

The genomic data landscape is rapidly expanding through three convergent forces: widespread adoption of clinical genetic testing (Phillips et al. 2018), large-scale population sequencing initiatives (e.g., UK BioBank, All of Us), (All of Us Research Program Genomics 2024; Bycroft et al. 2018) and advanced molecular assays that add rich biologic context to clinical data (Dai and Shen 2022). The next generation of AI systems in medical genetics will integrate multi-modal data to enable understanding of how genetic variants manifest differently across different organ systems and at the organism level, somatic and germline contexts, nuclear and mitochondrial genomes, development stages, and environmental conditions to generate functional insights with greater clinical relevance and impact (Garg et al. 2024). Language models for protein analysis (e.g., AlphaFold, AlphaMissense, ESMFold, and RosettaFold) highlight how genetic knowledge synthesis is advancing beyond sequence analysis by strengthening functional characterization with novel proteomic insights. These models can help explain functional characteristics of genetic disease by predicting how changes in amino acids affect both protein function and stability, while also evaluating pathogenicity (Baek et al. 2021; Cheng et al. 2023; Jumper et al. 2021; Lin et al. 2023).

When integrated with AI models that analyze protein–protein interactions and environmental factors, these tools enable refined interpretation of how variants affect cellular processes by disrupting protein networks, leading to different disease manifestations across various cell types (Abramson et al. 2024; Visscher et al. 2021). For instance, AI approaches can uncover patterns of digenic inheritance in disorders such as retinitis pigmentosa, where variants at separate loci collectively drive pathogenicity (Okazaki and Ott 2022). Furthermore, polygenic priority scores leverage these enrichments to predict causal genes in complex traits, while Bayesian networks model higher-order genetic interactions. Network-based strategies show promise for capturing disease complexity in settings where traditional Mendelian frameworks fall short (Howey et al. 2021; Weeks et al. 2023). Over the next two decades, AI will continue to accelerate the shift toward deep molecular phenomic profiling of disease to enable precision phenotyping at scale.

### Enhancing Phenotype- and Genotype-First Approaches

AI is also advancing genetic diagnostics through parallel enhancement of phenotype- and genotype-first approaches. DL tools like GestaltMatcher, which correlate facial features with genetic disorders, achieve high accuracy in ranking the correct disease gene within the top candidates, helping prioritize variants in exome sequencing analysis for patients with dysmorphic features (Hsieh et al. 2022; Schmidt et al. 2024). Natural language processing tools like FabricGEM analyze electronic health records (EHRs) to identify subtle phenotypic patterns indicative of genetic conditions to inform molecular testing in clinical settings (De La Vega et al. 2021). AI can also support systematic reverse phenotyping, or evaluation of individuals with specific genomic variants to identify associated phenotypes (Wilczewski et al. 2023). By analyzing genomic data via multiple disciplines, AI strengthens both diagnostic pathways, identifying genetic conditions from clinical features and expanding phenotypes associated with specific genomic variants. Furthermore, AI can enable continuous variant surveillance by integrating current real-world evidence into variant classification algorithms, as demonstrated by machine learning models trained on ACMG/AMP criteria to generate probabilistic pathogenicity scores for variants of uncertain significance, supporting evidence-based reclassification (Nicora et al. 2022).

### Designing Precision Therapies: AI-Guided Genetic Medicine

The emergence of disease-modifying genetic therapies is transforming medical genetics from a diagnostic to interventional field, with AI facilitating rational therapeutic design. DL models can predict off-target effects in CRISPR editing, optimize vector design for gene delivery, and generate synthetic regulatory elements with precise expression patterns with high performance (Capitanchik et al. 2024). Additionally, generative AI methods are enabling in silico testing of editing strategies, including designing synthetic promoters with tissue-specific expression patterns and predicting optimal guide RNA sequences for base editing applications (Dixit et al. 2023). Lab-in-the-loop approaches integrate these AI predictions with automated experimental validation platforms, creating rapid feedback cycles that continuously improve model accuracy and therapeutic safety (Chen et al. 2024). These advancements will enable the development of safer, more effective genetic therapies with unprecedented precision and efficiency.

### Navigating the Future: Ethics, Privacy, and Implementation Challenges

Despite immense promise, integration of AI in medical genetics presents specific challenges necessitating proactive solutions. Privacy-preserving methodologies must balance molecular phenomic data security with model performance (Gursoy et al. 2022; Yelmen et al. 2021). Validation standards require benchmarks for model performance across diverse populations, particularly for automated variant classification (Robson and Ioannidis 2024; Walton et al. 2024). Systematic bias detection in training data and regular model audits are essential, especially as algorithms influence clinical decisions, and clear thresholds for variant reclassification and model updates must be established (Mersha 2024; Yao et al. 2023) Biosecurity considerations are also paramount, requiring robust screening protocols to prevent misuse of AI-powered genetic design tools while preserving appropriate research access (De Haro 2024). As these technologies advance, medical societies must develop guidelines that provide validation frameworks to ensure clinical rigor as AI capabilities expand.

### Conclusion

Looking forward, AI will transform medical genetics from a primarily diagnostic discipline to an interventional one, enabled by dynamic representation of the molecular phenome and its functional and clinical significance. As genomic learning health systems continuously refine our knowledge through each patient interaction, these fundamental shifts will position geneticists as both diagnosticians and interventionists, reimagining genetics workflows across the patient journey.

## 3 | Next-Generation Phenotyping in the Global Healthcare System for Rare Disorders

### Tzung-Chien Hsieh

Next-generation phenotyping (NGP) technology, which utilizes facial image analysis to diagnose rare disorders (Gurovich et al. 2019; Hsieh et al. 2022), has advanced significantly over the last few years. Recently, the German National Rare Disorder Project (Schmidt et al. 2024) validated the clinical utility of GestaltMatcher (Hsieh et al. 2022) and Prioritization of Exome Variants by Image Analysis (PEDIA) (Hsieh et al. 2019) by showing performance improvement in predicting rare disorders with computational facial image analysis. With recent advances in sequencing technologies, exome sequencing (ES) and genome sequencing (GS) have become the diagnostic standard in many countries (see more specific discussion on AI as relates to laboratory-based analyses in the next essay, “AI in the clinical genomics laboratory”).

NGP technology will be a critical tool for interpreting the huge numbers of variants that are identified by these sequencing approaches, especially variants of uncertain significance (VUS). Looking ahead, we anticipate that NGP will be further integrated into healthcare systems, becoming a standard tool for diagnosing rare disorders. When the patient arrives at the clinic, similar to the way that trio-based ES or GS is conducted, their facial images (or other relevant image types) could be routinely analyzed, including, when possible, their parents’ images, enabling trio-NGP analysis. This may be particularly helpful to discern facial features that may relate to an underlying genetic condition from other, unrelated familial features. In this way, in low- and middle-income countries, where access to clinical geneticists and funding for ES or GS may be limited, trio-NGP could offer a cost-effective approach to guide genetic testing within constrained budgets.

In the future, NGP will extend beyond its current diagnostic use focused on individuals suspected to have a genetic condition to become a screening tool used in more general medical settings, enabling earlier detection of diseases. In the long term, NGP could be applied not only to newborn screening and routine pediatric health checkups after birth but also to non-invasive prenatal screening (NIPT) or other prenatal and peripartum diagnostics. Although prenatal screening typically relies on ultrasound images rather than standard frontal facial images, facial dysmorphisms can still be detected in fetal ultrasound scans. With advancements in ultrasound technology, image quality is expected to improve significantly, enabling more detailed analysis of fetal features. In cases where prenatal abnormalities are detected and the fetus does not survive, NGP can still assist in diagnosing the condition, providing parents with valuable insights to help inform future family planning decisions. However, for a variety of reasons, key challenges remain in fully integrating this technology into healthcare frameworks.

Privacy poses significant challenges to facial image analysis in rare disorder diagnosis, both technologically and culturally. Humans are easily identified by facial images. Developing secure infrastructures to store and analyze patient images while enabling data sharing within the research community is crucial for building diverse datasets and ensuring that these methods work broadly and equitably. Although the on-premise facial analysis solution can keep the patient’s image “in the clinic”, to ensure that NGP covers diverse populations, privacy-preserving methods such as Swarm learning (Warnat-Herresthal et al. 2021) which allows for decentralized model training without compromising individual privacy, is crucial. Therefore, the adoption rate of NGP will likely depend on connecting the global community with this type of decentralized approach.

Beyond technological measures, cultural considerations present another potential issue, as some parents may understandably hesitate to allow photos of their children to be used, fearing societal labeling or other harms. For example, this concern is particularly prominent in many East Asian cultures. A primary solution involves continuous conversation to build trust with patients and families by openly addressing their concerns and communicating the importance of facial analysis in achieving diagnoses. This trust also relies on secure healthcare systems that protect patient data and ensure privacy, ultimately encouraging broader participation in rare disorder research to help the larger community of those affected by genetic conditions.

Ultimately, the overall trend in the future will involve analyzing images from both probands and parents as a standard for both diagnostics and screening. With advancements in image quality and an inevitable shift from two-dimensional to three-dimensional imaging, NGP technology will become a more powerful tool for clinicians. As human clinicians cannot see and memorize the patterns of thousands of rare disorders, this will facilitate diagnosis and timely treatment. However, these ends can only be achieved when the global community works together and is supported by appropriate research and healthcare policies.

## 4 | AI in the Clinical Genomics Laboratory

### Tim Laurent, Maria J. Guillen Sacoto, Britt Johnson, Kirsty McWalter, Vincent D. Ustach, Paul Kruszka

Since the advent of next generation sequencing in 2007, the cost of sequencing the human genome has sharply declined, offering patients greater access to clinical genetic testing (see: https://www.genome.gov/about-genomics/fact-sheets/Sequencing-Human-Genome-cost). An increasing amount of clinical guidelines, payor policies, and, more recently, legislation, support GS and ES as the standard of care, indicating that the number of patients who could benefit from this testing will outpace the capacity of the geneticist workforce. AI provides an opportunity to scale to meet the needs of patient care. AI, recently popularized by products like ChatGPT, holds the promise of automating and augmenting the work of medical genetics professionals, allowing for increased efficiency, greater throughput, and enhanced quality of results for patients.

For this discussion, AI will include systems comprised of both narrowly trained ML models and generative LLMs. Narrow ML models are trained for a specific task, such as document classification (e.g., clinical notes vs. a genetic test report) or assessing the pathogenicity of a genetic variant. LLMs have been trained on internet-scale datasets to predict the next word and to generate outputs that can be helpful. These models can be applied to many diverse tasks, including generating human-like text and performing language-intensive tasks like document summarization. Modern AI systems often combine these approaches and integrate ML models with LLMs. An emergent pattern in AI involves *agentic* systems. Although a precise definition is hotly debated, an “agent” is often understood to be an LLM-based system with a repertoire of potential actions from which the system can choose to complete the given objective. Crucially, robust AI systems must be grounded in truth, often relying on knowledge bases to ensure that their outputs are factually accurate and reliable.

LLMs are trained on a massive corpus consisting of much of the internet and thus know a great deal about the world through this window. However, LLMs have knowledge limited to the data they were trained on up until their training date, and they do not possess information not included in their training set. This means they might not be aware of events, facts, or developments that occurred after their training cutoff or that were absent from their training data. Additionally, instruction-tuned models are trained to provide acceptable responses, and as a result, they may “hallucinate” or make up a reasonable-sounding, though incorrect, answer (Duong and Solomon 2023). One common approach to mitigate this in genetics is to use retrieval augmented generation (RAG), where relevant information is retrieved from a knowledge base and the LLM is instructed to use that information to answer the query.

LLM agentic workflows play a key role in high-performing modern AI systems. These are LLM-powered multistep programs where each step is an LLM with a specific role, and each agent can plan and use tools such as: retrieving information (e.g., a web search or querying an internal database); calling a function (e.g., performing a mathematic operation); calling other agents (e.g., consulting with or delegating tasks to other agents); or outputting structured information. This allows for complex workflows to be broken into simple parts and can better solve problems than a single-shot LLM call (Gou et al. 2023).

While there are many ways that AI can enhance the business of genetic testing, such as automating billing and for customer support, AI’s potential in genetic interpretation is particularly compelling. AI uses within medical genetics include automating aspects of the clinical workflow, providing predictions of patients’ genetic conditions, and deriving insights from the cumulative testing dataset, including uncovering new gene–disease relationships.

An important aspect of AI in medical genetics is the user experience or interface that operationalizes it. It is important for clinical staff to be able to view the AI predictions and the provenance for how the predictions were determined. For example, Human Phenotype Ontology (HPO) terms extracted from clinical documents should be verifiable to the original source, and it should be easy for the clinician to correct any erroneous predictions or add new terms. This will provide important feedback to the system to improve it either through fine-tuning or as part of an evaluation dataset. The human-in-the-loop pattern enables AI to greatly enhance the efficiency of clinical work while maintaining high accuracy and oversight of the AI predictions.

The workflows of a genetics laboratory include analysis and extraction of features such as HPO terms from clinical documents, review of the genetic literature, assignment of American College of Medical Genetics (ACMG) criteria to variants (Richards et al. 2015), and generation of a test report to communicate results to patients and clinicians. In all of these use cases, AI can provide solutions to perform tasks more reproducibly, more thoroughly, and faster. For example, conventional or generative AI can be used to extract structured clinical information from clinical documents (Fu et al. 2024; Srivastava et al. 2024), mine the genetics literature for evidence attributable to the variants (Lyu et al. 2024; Wei et al. 2018), and pull together relevant test information and information from the literature to draft a genetic test report. AI has been used for similar tasks within other knowledge-intensive fields such as legal (e.g., https://www.clio.com) and software (e.g., https://www.cursor.com) realms. The summarization and synthesis of genetic findings from many publications and internal and external cases can be enhanced by agentic workflows that generate knowledge-grounded first drafts of passages to consider for inclusion in the final patient report.

Identifying relevant variants and determining their pathogenicity is another extremely important part of the workflow that can be powered by AI. This is a process of whittling thousands of potential variants down to one or several that are responsible for the patient’s clinical presentation. There has been great advancement in DNA sequence models like ESM-var (Meier et al. 2021) and protein-sequence models such as AlphaMissense (Cheng et al. 2023) that can predict pathogenicity of genetic variants. Additionally, knowledge-based models of disease such as SHEPHERD (Alsentzer et al. 2024) provide powerful tools for recommending the causative variant or gene in a particular patient’s case. Many of these methods rely on clever strategies to find data upon which to train, for example, population frequency variant data in the case of AlphaMissense or “synthetic patients” for SHEPHERD. Large commercial laboratories have robust patient datasets of ES and GS data paired with detailed clinical information for patients suspected of rare disease, which can also be used to train highly effective models of variant pathogenicity.

In addition to helping to solve a single genetic case, AI can leverage large public and commercial genetic datasets that couple genotype and phenotype information to extend the boundaries of knowledge and find answers for more patients. AI models can integrate the mountains of genomic data to find new gene-disease candidates, help understand pathogenic non-coding variants, and generate models of polygenic risk (Greene et al. 2017; Wells et al. 2019).

The future is bright for AI in medical genetics. The challenge will be to deploy it in a reliable, factual, ethical, compliant, and human-centric way to ensure quality of care for patients.

## 5 | Future of AI and Medical Genetics and Genomics Education

### Rebekah L. Waikel

Despite the prevalence of genetic conditions, there is a lack of standardized genetics training in medical school, residencies, and beyond. This issue, along with the shortage of medical geneticists, necessitates innovative solutions such as the use of AI to train clinicians, including to recognize as well as manage genetic conditions. While AI methods have been successfully used in many clinical specialties for educational purposes, particularly radiology and surgery (Gordon et al. 2024), these approaches are not yet in mainstream use in medical genetics. Recent studies highlight the need for more AI education in medical genetics, as well as the promise of DL tools to help with tasks like dysmorphology recognition (Hsieh et al. 2022; Waikel et al. 2024) Here, we succinctly describe examples of some recent and emerging studies that help highlight the need for, as well as the potential of, this type of AI application in medical genetics.

A new survey of the attitudes and perceptions of the current clinical genetics workforce, which included 215 US-based genetics clinicians and genetics trainees, found that over half (51.4%) of participants report little to no knowledge of AI in clinical genetics and 76% reported no formal training in the use of AI tools in this context (Berkstresser et al. 2025). Formal training directly correlated with self-reported knowledge of AI in clinical genetics, with 67% of respondents with formal training reporting intermediate to extensive knowledge versus 39% without formal training. Nearly all participants (97.6%) indicated that more AI education is needed for use in clinical care, with 89.3% reporting they are willing to take a course about AI in clinical genetics, if available. The results of this survey demonstrate both the need and desire among clinical genetics professionals for more clinical-based training in AI.

To date, there is one published case study of AI and clinical genetics education, which explored the use of generative AI images to train pediatric residents to recognize facial images of children with two genetic conditions, Kabuki (KS) and Noonan (NS) syndromes (Waikel et al. 2024) Generative adversarial networks (GANs) were used to make synthetic but realistic images of children with these conditions, as well as images showing a child transforming from an unaffected to an affected child (this type of image, based on studies showing disease progression in other genetic conditions (Duong et al. 2022), had been anecdotally suggested as helpful for learning dysmorphology by trainees). Pediatric residents were more familiar with NS, as evidenced by baseline accuracies for recognizing NS (65.3%), with just a text description compared to 48.2% for KS. All image interventions (real and synthetic) provided some improvement in accuracy, particularly for KS images, with a 12.1% improvement between text only and text plus real KS images. A similar improvement was observed with the generative images showing the transformation of an unaffected synthetic individual into an individual with KS (11.4% improvement) and a single set of synthetic KS images (8.8%). Accuracies for NS images with the addition of real images, synthetic images, and transformation images were 74.3%, 68.0%, and 71.0%, respectively. Both real images and synthetic images provided improvement over text-only descriptions of genetic conditions, particularly for rarer conditions like KS, suggesting that synthetic images can supplement existing real images. Synthetic images provide potential advantages over real images, including addressing privacy concerns, particularly for minors and other individuals who are unable to provide consent. Additionally, these images can be generated to include more ancestral and other types of diversity than existing textbook images. Both GAN and diffusion models continue to advance, enabling the generation of more realistic synthetic images as well as images that can be made more specific to a given user prompt (Veturi et al. 2023).

Another area of interest is whether studying expert professional and computer vision and visual attention can provide insight into novel approaches to train clinicians to recognize important features in genetics, such as dysmorphic features that can help with the differential diagnosis of genetic conditions. “Professional vision” has been well characterized in teacher/classroom settings to identify critical features that relate to teaching expertise. In brief, two interconnected processes have been studied using eye tracking technology: (1) noticing (the ability to direct attention to relevant classroom events); (2) knowledge-based reasoning (the ability to interpret these events and anticipate consequences for further learning) (Gegenfurtner et al. 2019; Goodwin 1994; Seidel and Stürmer 2014). Expert teachers were found to have shorter fixation durations, more task-relevant fixations, and fewer fixations on task-redundant areas (Wolff et al. 2016). Similar analyses may be helpful in medical genetics. For example, Duong et al. recently published a study comparing the heat maps generated by eye tracking experiments of expert clinical geneticists viewing images of people with and without genetic conditions with computer vision-based results (Duong et al. 2024). Human experts demonstrated similar gaze paths for a given syndromic facial image, which was different from that of a computer model’s pixels of interest as determined by a saliency map, a type of explainable AI (xAI). An interesting extension of this work will be to determine whether human expert eye tracking maps and saliency maps can be used together to teach and improve recognition of genetic conditions.

In summary, there is interest among the clinical genetics community to gain more experience with AI tools, and early studies in or related to this area show promise. We recommend that AI tools be further explored for medical education. Future projects likely will involve (1) investigating other generative methods, including those related to more realistic images, more genetic conditions, and other types of transformative images; (2) utilizing expert eye tracking to teach facial examination, as well as (3) xAI outputs, such as saliency maps, to teach dysmorphology and syndrome recognition. Other non-image-based generative AI education tools could include using LLMs to generate medical genetics vignettes and patient/clinician conversations (Flaharty et al. 2024; Othman et al. 2025). AI tools provide novel approaches that may augment genetics education, can be implemented in ways that ameliorate the national and international lack of medical genetics educators, and can be a window to help clinicians become more familiar with the use of AI tools.

## 6 | Using AI to Realize Data-Driven Definitions for Genetic Diseases

### Melissa A. Haendel, Yousif J. Shwetar

A crucial but often overlooked challenge in medicine is establishing clear definitions for diseases. These definitions serve as reference standards against which we compare individual patients when making diagnoses. Without precise disease definitions, the diagnostic process becomes less reliable. Historically, medicine has defined the classification of disease (nosology) based on clinical phenotypes observed in patients and families. Precision medicine, however, requires comparing the patient’s features against a much more granular representation of disease knowledge; examples include the obvious associated genes and variants, but also underlying mechanisms, the spatiotemporality of the phenotypes, ‘omics signatures, and environmental factors (Haendel, McMurry, et al. 2018). Such information has proven challenging to reconcile reliably across knowledge sources. In the context of rare genetic diseases, there is an even greater challenge as case-level patient attributes are needed to create the reference knowledge others need to make diagnoses.

While many disease terminologies exist, there is no established standard for disease coding that fully supports information exchange needs. For example, no terminology covers all disease concepts needed for fine grained clinical coding, such as rare genetic diseases (Vasilevsky et al. 2022). Existing sources of disease definitions include the ICD-9/10, National Cancer Institute Thesaurus (NCIt), OMIM, Orphanet, SNOMED CT, MedGen, and many others. Each of these terminologies is designed for a particular purpose, and as such has different strengths. However, these standards only partially overlap and often conflict in their classification and/or mapping to identical disease concept terms across resources (Haendel, Chute, et al. 2018). Additional differences include disease naming conventions, synonym encoding, and cross-referencing (mapping). The need to integrate information has resulted in a proliferation of mappings between disease concepts in different resources; however, these mappings lack completeness, accuracy, and precision, and are often inconsistent between resources, rendering them inadequate for use in many settings, including precision medicine. Most importantly, none of these resources are directly informed by individual case-level patient attributes. Medicine has matured to the point where clinicians and researchers can no longer rely on diagnostic criteria existing as lists of inclusion/exclusion features. Therefore, there are three main trajectories for artificial intelligence (AI) to aid in the definition of diseases as reference knowledge to inform diagnostics, as follows.

First, AI is becoming increasingly useful to reconcile disease definitions across knowledge sources. This opportunity leverages “good old-fashioned AI” with semantics and reasoners, together with opportunities afforded by LLMs. The correspondence (mapping) among individual concepts is often accomplished through text-matching, but this can be misleading and potentially dangerous; for example, the terms ‘Muscular pseudohypertrophy-hypothyroidism syndrome’ [Orphanet: 2349] and ‘B cell immunodeficiency-limb anomaly-urogenital malformation syndrome’ [OMIM: 609296] both have the synonym ‘Hoffman syndrome’, but they are entirely different diseases. Because of these challenges, resources providing disease definitions are often incomplete, inconsistent, and unreliable—especially collectively—whether for diagnostics or research. Using a combination of graph-based computational inference and LLM extraction of disease-associated content from databases and the literature, we can align disease definitions, collate evidence for definitions and “lumping and splitting” of specific diseases (Thaxton et al. 2022), and have the AI tool make suggestions for reconciling conflicts. The result is a human-in-the loop system using multiple AI approaches to create a consensus of disease definitions that can be readily and regularly updated and enhanced as new knowledge becomes available.

The second aspect of using AI to help define diseases is the incredible opportunity to engage patients in obtaining case-level data. Historically, genetic counselors and clinical geneticists have taken family history in paper files and pedigrees, and the associated data themselves have been barely even electronic. The literature is full of case reports that have cohort-level tables about patient features, but not the specific features associated with individual patients. This lack of granularity has led to a significant gap in understanding incidence, expressivity, and penetrance of patient phenotypic features. Once these case-level details are available, AI and machine learning (ML) tools can take advantage of these data to weight diagnostic probabilities in a much more sophisticated manner. Patients are a great source of information about their own genetic diseases—they live them all day, every day. Patients experience things that are not easily observed clinically, such as a baby that has no tears (Enns et al. 2014). AI tools are especially useful to garner new insights from patients and can be tasked to interact with them in ways that are much more versatile, clever, and faster than traditional survey methods.

A combination of AI tools can allow a patient to text or speak with a system that can prompt specific questions in response to a patient’s description of their symptoms. For example, an AI bot might respond to a patient documenting patchiness of skin pigmentation by asking about their cancer or diabetes history based on associations with Fanconi anemia. In this example, there is an LLM talking to the patient, a backend knowledge graph containing information about genetic diseases (e.g., Translator (Fecho et al. 2022) or Monarch (Putman et al. 2024)), and a large cohort of patient-level data within an EHR system and/or using case level descriptions such as those found in Phenopackets (Jacobsen et al. 2022). The combination of LLMs and use of graph AI methods together could expedite the patient-level information and could solicit specific information from the patient much more quickly than the standard intake survey; however, care must be taken not to overly “lead” the patient on and ensure that all body systems are considered (e.g., see: https://www.npr.org/sections/health-shots/2023/06/08/1180838096/an-eating-disorders-chatbot-offered-dieting-advice-raising-fears-about-ai-in-hea).

Finally, a major opportunity involves integrating multimodal patient-generated and clinical data. With patient consent, data from wearables and photographs could be included alongside the aforementioned interactive phenotyping and could be combined with a variety of multi-omics data. For example, in Stargardt disease, Convolutional Neural Networks (CNNs) have demonstrated subpixel accuracy in the task of optical coherence tomography (OCT) retinal layer segmentation (Kugelman et al. 2020; Mishra et al. 2021), while clinical studies have shown that relatively simple phenotypic characteristics such as foveal sparing and age of onset are useful for assessing disease severity (Fujinami et al. 2015; Rotenstreich et al. 2003; Testa et al. 2014). While long read sequencing, transcriptomics, tissue imaging, cytokine profiling, and epigenomics are still relatively unavailable to most diagnosticians, it will be the advances in integrative AI technologies that will popularize their use in mainstream settings, bringing genomic healthcare to non-expert contexts.

By harnessing the synergy of AI and diverse data streams, we can create a richer picture of each individual patient. This deeper understanding of all patients not only sharpens our diagnostic precision but creates a powerful foundation of knowledge that continuously evolves our definition of diseases. Sharing these refined disease definitions across borders transforms genomic healthcare from a privilege to a standard of care, saving countless lives while dramatically reducing both costs and time to treatment.

## 7 | Strategies for AI in Medical Genetics: Rare Diseases and Small Datasets

### Dat Duong

Within the past decade, DL classification and generative methods for image and text datasets have improved tremendously and proven to be extremely valuable in the field of medical genetics. In some instances, it is possible to obtain very accurate answers related to medical domains from generalized models trained on data from sources like internet websites and other text resources (e.g., models that are not trained specifically on just medical datasets) (Flaharty et al. 2024).

However, there remains a major challenge related to training and using deep learning methods: the lack of accurate, human-labeled datasets. While this challenge pertains to all areas of deep learning, it is especially relevant to medical genetics. This stems from the rarity of individual genetic diseases and the lack of trained experts in these diseases. To explain this, we could use the analogy of genomic sequencing to find the cause of a person’s medical condition. Genomic sequencing will be most useful when it is able to detect a wide range of different genetic causes of medical conditions, including conditions related to different genes, different molecular mechanisms (e.g., single nucleotide variants, structural variants, repeat disorders, etc.). To be able to detect all such conditions, the genomic sequencing assay has to be trained and validated appropriately.

Deep learning faces an analogous issue—to be able to detect (or otherwise analyze) a very broad range of conditions, there must be adequate data. One straightforward solution is to simply amass more datasets for these rare diseases (Lesmann et al. 2024) but this can be very time-consuming, extremely costly due to requirements such as the need for expert annotators, and may still face challenges related to data scarcity for many conditions despite such efforts (Hsieh et al. 2022).

However, there are other ways to address the small sample size problem, and some promising strategies are just starting to emerge. In the future, these strategies may make deep learning an even more useful tool for the field of medical genetics. One common approach is to leverage existing large datasets that are closely related to the much smaller medical datasets. For example, in a recent paper on the use of LLMs to identify genetic conditions based on short text descriptions, the authors did not need to train a LLM on their own small datasets (Flaharty et al. 2024). Instead, the authors used OMIM (https://www.omim.org/) to retrieve genetic conditions with clinical features that were similar to those in their own dataset. This retrieved information was used as in-context data for the LLM, which then enabled the LLM to predict the possible diseases more accurately for the conditions described in their small datasets.

As another example, in a study on how different variables (in this case, facial expression) affect the ability of a deep learning model to identify genetic conditions based on facial images (Patel et al. 2024), the authors combined a smaller face image dataset (consisting of images of people with the genetic conditions under investigation) with a larger, general facial image dataset. Then, a deep learning image generator was trained on this larger, aggregated dataset. The authors were then able to alter the expression for the faces of people with genetic conditions from the smaller datasets via the image generator. The outcome here was superior to those of an image generator trained using only smaller datasets. Other similar examples, in which larger datasets were used along with the smaller targeted datasets, include deep learning models involving studies about genetic conditions with cutaneous features (Duong et al. 2022), foveal hypoplasia (Malechka et al. 2023), and rare diseases with observable facial syndromic features using few/zero shot learning (Hsieh et al. 2022).

Another approach is to modify and train only a small fraction of the parameters in a foundational deep learning model (e.g., a model that has been trained on a very large dataset that may or may not be related to medical genetics). By lowering the number of trainable parameters, one may obviate the need to use a very large dataset. One example involves the application of segmentation methods on MRI images (Gu et al. 2024). Here, along with various other training techniques, the authors also evaluated low-rank adaptation, which decomposes large matrices of a deep learning model into smaller matrices, and thus reduces the number of trainable parameters.

Hence, in the medium term of 10–20 years, as data is continually being amassed, there will be more available large datasets that can be used along with smaller medical genetics datasets in order to train deep learning models. Researchers can take advantage of the types of approaches outlined here, but building accurate models may become easier. For example, an annotated and high-quality dataset of vertebrae positions of cervical X-ray images was recently released (Ran et al. 2024). This is helping a current research project that involves using deep learning to analyze X-rays for a specific genetic condition, for which a relatively small dataset is available due to the rarity of the condition. Moreover, as methods like low-rank adaptation are continually being developed and explored in the context of medical genetics, training a large deep learning model should become more manageable for smaller datasets. That is, more ingenious approaches may be devised that help ensure that deep learning models can work accurately for small datasets consisting of rare conditions as for common conditions, where dataset size is not as challenging. We then would expect deep learning to be implemented in many more situations than it is today where sample size is still lacking. This should directly translate to clinical applications. For example, AI models that aim to provide differential diagnoses based on facial images will be more useful when they can recognize more possible conditions rather than a subset. Advances should also help with the recognition of the range of presentations of conditions, such as in people of different ages, sexes, ancestries, with different clinical manifestations.

## 8 | Artificial Intelligence for Diagnosing and Monitoring Genetic Bone Diseases

### Behnam Javanmardi, Klaus Mohnike, Peter Krawitz

Genetic bone diseases (GBDs) are a diverse group of disorders marked by characteristic abnormalities in bone and cartilage. In approximately the past decade, the number of known GBDs has increased from 436 (associated with 364 genes) in 2015 (Bonafe et al. 2015) to 771 (associated with 552 genes) in 2023 (Unger et al. 2023), highlighting progress in identifying new disorders at the molecular level, which was largely made possible by advancements in DNA sequencing technologies. During the same time, an increasing number of precision therapies had been developed (Aartsma-Rus et al. 2021; Demirci et al. 2024). Although many GBDs still have no known cure, most patients will eventually require complex, multidisciplinary care and lifelong management, often involving surgeries (Tosi et al. 2020).

Timely diagnosis is essential for tailoring appropriate care and treatment for people with GBDs. General practitioners, such as family physicians and pediatricians, are often the first point of contact for such patients, though these generalist clinicians typically lack experience in diagnosing, counseling, and managing patients with rare diseases like GBDs. Further, the growing number of conditions with known genetic causes makes it very challenging even for experts to keep track of the literature. Despite the availability of genetic testing, the diagnostic journey for many patients can still take several years (Hay et al. 2022), and around 50% of GBD cases remain undiagnosed (Sabir et al. 2021), including due to challenges related to the interpretation of the numerous VUS that may be detected through sequencing. This highlights that genetic testing alone is not sufficient; establishing a connection between genotype (molecular data) and phenotype (perhaps especially related to radiologic imaging) is crucial for accurate diagnosis (Handa et al. 2023).

Already in 2012 (Hennekam and Biesecker 2012), leaders in the field posited that “next-generation sequencing demands next-generation phenotyping” and that novel approaches for detailed phenotyping should be integrated with sequencing results. Since then, AI methods (in particular those involving computer vision) have been shown to be very helpful in accelerating the diagnosis of rare neurodevelopmental diseases, such as by assessing facial features (Gurovich et al. 2019; Hsieh et al. 2022), and rare eye diseases, such as by assessing ophthalmologic imaging (Pontikos, Woof, et al. 2022). For more on these topics, see the essays entitled, “Next-generation phenotyping in the global healthcare system for rare disorders” and “Perspectives on the future use of artificial intelligence in inherited retinal diseases”.

The same principle described above is at the foundation of the Bone2Gene AI (https://bone2gene.org, currently under development). This has been motivated by interest from the community of clinicians and researchers who support and study GBDs. In a recent global survey conducted by the authors (Javanmardi, Waikel, et al. in preparation), over 80% of the participants, regardless of years of experience, reported that the delineation between different GBDs based on visual inspection of patients’ radiographs is somewhat or extremely difficult and that it is somewhat or extremely likely that they integrate an image recognition AI in their current diagnostic workflow.

Bone2Gene will learn to recognize and differentiate the characteristic imaging patterns associated with different GBDs. In the first phase, the Bone2Gene AI will be trained on dorsopalmar hand and wrist radiographs from individuals with different GBDs. This choice was made because obtaining a hand X-ray for bone age assessment is a routine procedure for children suspected of having bone abnormalities (Rassmann et al. 2024). In the next phases, Bone2Gene will be expanded beyond the hand and will be trained on radiographs of other body parts (such as the spine, hip, etc.).

Whether deployed at radiology labs or clinics, Bone2Gene is anticipated to improve the diagnostic journey at three different stages or areas of expertise: (a) general clinicians, such as family physicians and pediatricians, (b) specialists (e.g., orthopedists, endocrinologists, and other specialists involved in the care of patients with GBDs), and finally, (c) medical geneticists. In general, in the first two stages, though uses may vary based on a specific user, Bone2Gene can accelerate the detection and referral of GBD patients. In the last stage, Bone2Gene can assist with the interpretation of the genetic test results and the differential diagnosis.

Furthermore, Bone2Gene will also be trained on longitudinal data at different stages of disease progression, hence assisting with ongoing monitoring of GBDs. This is especially crucial for disorders with available therapies (such as vosoritide for achondroplasia) (Chan et al. 2022; Savarirayan et al. 2024), where assessing drug response and determining treatment suitability and continuation is essential. These efforts can thus contribute to clinical trials as well as surveillance related to approved therapies.

The overarching goal of the Bone2Gene AI is to become a reliable assistant for non-geneticists as well as medical geneticists in diagnosing GBDs. Alongside other AI-based phenotyping tools, Bone2Gene aims to shorten the diagnostic odyssey, which, in turn, would reduce the overall costs of the diagnostic process for healthcare systems and society and alleviate challenges faced by patients and families affected by rare diseases.

## 9 | Perspectives on the Future Use of Artificial Intelligence in Inherited Retinal Diseases

### Thales A. C. de Guimarães, William Woof, Michel Michaelides, Nikolas Pontikos

#### Background

Inherited retinal diseases (IRDs) are among the leading causes of severe visual impairment in the working age population and in childhood (Heath Jeffery et al. 2021; Liew et al. 2014). The diagnosis of these conditions relies heavily on multimodal retinal imaging (Georgiou et al. 2024), which generates huge amounts of data. In fact, it is estimated that approximately 30 million retinal optical coherence tomography (OCT) scans are acquired yearly in the USA alone (Pontikos, Wagner, et al. 2020; Ting et al. 2020), a scale and type of data that provide an optimal environment for AI-derived tools to thrive. Other imaging modalities that foster the development of this area, particularly in the field of DL, include fundus autofluorescence (FAF) and color fundus photographs (CFP).

The latest advances in the field of AI have generated refinement in artificial neural networks and DL to identify characteristics in images relevant to ophthalmology. These models can be trained to identify features of specific IRDs from a variety of imaging modalities. These tools are currently being used for the purposes of classification, identification/diagnosis, genotype prediction, genotype–phenotype correlation, and prognostication of IRDs (Chen et al. 2021; Fujinami-Yokokawa et al. 2021; Miere et al. 2020). Selected key studies related to these topics will be discussed herein.

#### Applications of AI in IRDs

##### Phenotype Identification.

Arsalan et al. (2020) developed a fully convolutional network (RPS-NET) that was able to segment and detect intraretinal pigments in CFPs of patients with retinitis pigmentosa (RP) (these intraretinal pigments are a cardinal feature of RP), achieving an accuracy and specificity of 99.5% and 99.6%, respectively (Arsalan et al. 2020). Subsequently, Chen et al. (2021) used a DL model to detect the presence of RP through analysis of CFPs, the results of which were compared with four ophthalmologists with varying degrees of experience in IRDs (Chen et al. 2021). The model achieved accuracies as high as 96% versus an average of 81.5% for the four ophthalmologists, with the same level of sensitivity (95.7%). Another group developed a CNN to differentiate OCT images of healthy controls from patients affected with Stargardt disease (STGD), achieving an accuracy of 99.6% when implemented as a binary classifier (Shah et al. 2020).

##### Genotype Prediction.

Given the growing availability of large datasets of IRD patients with both genetics and imaging data available (Pontikos, Arno, et al. 2020), the vast majority of studies currently focus on genotype prediction, with several CNNs that have been developed for a variety of purposes Miere et al. (2020). used a CNN to distinguish FAF images of patients with RP, Best disease (BD), and STGD, with global accuracy for IRD classifiers of 95% and precision-recall area under the curve averaging as high as 99.9% for RP (Miere et al. 2020). The same group subsequently assessed the performance of a pre-trained CNN in differentiating *ABCA4*- and *PRPH2*-associated retinal dystrophies, diseases which share many similarities (Georgiou et al. 2024), with a good accuracy that surpassed retina experts (Miere et al. 2021). Similar models were also employed in OCT with or without FAF images to predict conditions, such as (i) *ABCA4-*, *RP1L1-*, and *EYS*-associated retinal dystrophies (Fujinami-Yokokawa et al. 2021, 2019), and (ii) BD versus adult-onset vitelliform macular dystrophy (Crincoli et al. 2022).

The use of multimodal imaging has been expanded to differentiate 36 genes in the Eye2Gene project, a work led by Pontikos et al. achieving a top-5 accuracy of 85.6% (Nguyen et al. 2023; Pontikos, Woof, et al. 2022).

##### Monitoring of Disease Progression.

Wang et al. (2019) employed DL for automated screening and segmentation of atrophic lesions in FAF images of patients with STGD, with an accuracy of 95% in differentiating healthy eyes and those with STGD (Wang et al. 2019). Another group validated a DL-derived segmentation tool in FAF for detecting and measuring changes in hyperautofluorescent flecks of patients with STGD (Charng et al. 2020). The authors reported good overall agreement between manual and DL segmentation in both fleck area and fleck count in three concentric circles of 10°, 20°, and 30° diameter. More recently, Woof et al. used manually annotated FAF features to produce segmentation masks with the purpose of training an AI model—called AIRDetect—on a sample of 45,749 images from 3606 patients affected with IRDs (Woof et al. 2024). This work allowed for quantitative analysis, both cross-sectionally and longitudinally, of FAF features in a wide range of IRDs.

#### Conclusions

AI has now become a reality in the IRD field. Many research groups are actively adopting these tools for a variety of applications, some of which have direct implications in clinical trial endpoint assessment, patient stratification, patient prognostication, and diagnostic purposes. The future is incredibly promising, and we remain highly optimistic that AI is on the verge of revolutionizing healthcare for our patients with IRDs.

## 10 | Prospective for AI Applications in Medical Genetics in Taiwan

### Ni-Chung Lee

#### Introduction

Just as AI should not be thought of as a single entity, there are many different ways to study and implement AI. Research and clinical uses of AI are profoundly affected by factors such as local, regional, national, and international regulations, practices, and resources, and it can be instructive to take a deeper dive into examples of this, similar to the way in which some of the essays in our previous collection shed light on how dysmorphology is practiced in different parts of the world (Solomon et al. 2023). With this in mind, we turn to Taiwan, which presents a fascinating lens into this question in a geographic region that is in many ways at the AI epicenter.

Taiwan, renowned for its technological prowess and robust semiconductor industry, is uniquely positioned to leverage AI for advancements in medical genetics. Despite challenges such as a smaller patient population (Taiwan has a population of ~23 million on a 36,197 km^2^ island) and talent scarcity, Taiwan’s strengths in advanced technology, particularly with companies like the Taiwan Semiconductor Manufacturing Company (TSMC), and proactive government strategies provide a fertile ground for AI advancements in many different fields of application.

Taiwan’s robust hardware industry provides a solid foundation for the development and deployment of advanced AI algorithms and computational models essential for AI deployment. On the software front, Taiwan has developed multiple AI-driven tools for healthcare. These tools facilitate rapid and accurate interpretation of patients’ data, which is crucial for diagnosing disease. Overall, the integration of AI methods into analytic software enhances the diagnosis of specific conditions, which in turn leads to improved patient outcomes.

#### Government Support and Strategic Initiatives

The Taiwanese government has been proactive in supporting the integration of AI in healthcare. Since 2017, the Ministry of Science and Technology (MOST), now the National Science and Technology Council (NSTC), launched the Artificial Intelligence Innovation Research Center Program, marking the formal start of the AI era in Taiwan. Since then, various funding opportunities have been available for researchers and institutions working on aspects of AI applications in healthcare. The government’s commitment to building a smart healthcare ecosystem provides a conducive environment for the adoption of AI in healthcare. The Joint Commission of Taiwan also launched a Smart Healthcare accreditation in 2013, which facilitates the incorporation of advanced technologies, including AI, into Taiwanese hospitals in order to enhance medical services and patient care.

Due to this type of governmental support, Taiwan’s industry has been actively contributing to the AI landscape in health care. Several companies and research institutions are developing innovative AI solutions for diagnostics. For example, AI algorithms for bone age assessment have shown promising results in clinical settings, demonstrating the practical applications of AI in healthcare (Cheng et al. 2022). Currently, eight AI-powered medical products have been registered with the Taiwan Food and Drug Administration (see: https://lmspiq.fda.gov.tw/web/MDPIQ/MDPIQLicSearch). Based on a review of PubMed (date of review: November 2, 2024), there are currently 441 research papers from Taiwan on AI applications in healthcare, representing a wide variety of relevant applications. These publications highlight the advancements and innovations emerging from Taiwanese researchers and institutions, underscoring Taiwan’s active participation in the global AI research community.

#### Applications in Medical Genetics

Applications of AI in genetics in Taiwan can be divided into clinical and laboratory categories. For clinical applications, facial recognition apps (e.g., Face2Gene and GestaltMatcher) have been well-adapted in daily practice in clinical genetics in order to help suggest potential diagnoses based on facial features (Gurovich et al. 2019; Lesmann et al. 2024). Although many Taiwanese parents hesitate to have their children’s photos taken, and the diverse facial features of Asian patients and variable representation in training datasets may reduce accuracy, the increasing size of the databases is expected to address these issues soon. For laboratory applications, a main area of interest lies in developing AI-powered NGS diagnostic tools. With the support of MOST, two groups in Taiwan had developed AI-powered tool for variant prioritization (Huang et al. 2022; Lin et al. 2021). This has been incorporated into clinical NGS (exome or genome) testing as well as into developing models that could be used in more limited genetic testing settings. This use of AI is particularly crucial in Taiwan, where the government supports initiatives that can be used for the early diagnosis and treatment of genetic diseases. For example, there are currently 47 research papers from Taiwan on AI applications in medical genetics; these largely involve international collaborations and focus on polygenic diseases. However, we foresee that the future use of AI in for medical genetics will produce tools that can be globally applicable, both in terms of the types of conditions as well as populations of patients. See the essay entitled, “Next-generation phenotyping in the global healthcare system for rare disorders” for more on this point.

#### Challenges and Limitations

Despite the history and future prospects of governmental support, Taiwan faces several challenges in fully realizing the potential of AI in medical genetics. One of the primary challenges is the relatively small patient population, which limits the diversity of genetic as well as clinical data available for AI training and validation. This limitation can affect the generalizability and accuracy of AI models. Another concern is the conservative attitude that many people in Taiwan hold regarding the use of healthcare data (e.g., electronic health records) for model training, cloud utilization, and other such uses that are important in AI. Currently, stringent regulations by the government and Institutional Review Board Committees often slow down AI model development in Taiwan. In this circumstance, governmental actions may be necessary to help encourage data to facilitate AI training and genomic studies.

#### Conclusions

Taiwan is well-positioned to leverage AI in advancing medical genetics, thanks to its strong technological infrastructure, government support, and ongoing research efforts. Continued incorporation of available AI tools in Taiwan, coupled with strategic initiatives by the government, will be crucial in overcoming these challenges and harnessing the full potential of AI in medical genetics. However, one limitation comes from the stringent regulation; in order to incorporate AI into medical genetics, the government will play an important role. To return to the introductory statements, it will be important to consider the incorporation of AI in medical genetics in multiple regions of the world, including because global cooperation is likely to become increasingly critical in order to best serve patients, research participants, clinicians, and researchers (Lesmann et al. 2024).

## 11 | Flipping the Hierarchy: AI in Medical Genetics Research

### Benjamin D. Solomon

The field of medical genetics has always been deeply intertwined with research. Clinical practice goes hand-in-hand with discovering disease causes, defining the sequelae of genetic variants, and developing new therapies and management approaches. The connection between clinical practice and research in medical genetics seems unmatched in other realms of medicine. As a result, medical genetics feels dynamic and cutting-edge and can offer future hope even in the face of present-day dead-ends. Due to technological improvements, the parents of a currently undiagnosed child may soon be offered a precise explanation for her medical condition (Torene et al. 2020). Data sharing platforms have allowed much better understanding of the natural history of many disorders (McWalter et al. 2022). Though diagnostic advances have admittedly outstripped the availability of treatments, advances in gene therapy, gene editing, and other pharmacologic interventions offer great promise (Green et al. 2020).

It seems crystal clear that AI will fundamentally alter clinical aspects of medical genetics, as well as the entire practice of medicine. Exactly how this will unfold is—at least to me—hazier (Solomon and Chung 2023). With this in mind, let us turn our attention away from the clinic and toward research. I want to briefly focus on at least one major (perhaps earth-shattering would be a better term) way that I believe AI will upend research, which again plays such a central role in medical genetics.

Medical genetics research depends heavily, and increasingly, on computational methods. These methods are often undergirded by different types of AI (whether researchers are aware of this or not!). For example, researchers might use programs like SpliceAI (Jaganathan et al. 2019) DeepVariant (Poplin et al. 2018), or AlphaMissense (Cheng et al. 2023) to analyze genomic data and assess genetic variants. They might use tools like Phenomizer (Kohler et al. 2019) or leverage the GestaltMatcher database (Lesmann et al. 2024) to correlate phenotypic findings with specific genetic changes (Hsieh et al. 2022), or use machine learning models to assess gene expression when searching for biomarkers or trying to tease out the underlying biology of a medical condition (Schmauch et al. 2020). Many powerful computational models are available; some are slickly packaged as off-the-shelf solutions, while others require programming knowledge for implementation, or may support the integration of AI-based methods by sophisticated computational researchers. All of these currently have in common the fact that they are human-directed. That is, clever and passionate human researchers can use these and many other computational tools to make their investigations more accurate, efficient, and cost-effective. These tools are especially suited to combing through massive, often messy datasets to unearth subtle but important findings humans might never notice. As AI models became more powerful and more ubiquitous, medical genetics research has yielded fascinating results, including the ability to very accurately identify different genetic conditions from images like faces (Hsieh et al. 2022), ophthalmologic images (Pontikos, Woof, et al. 2022), and X-rays (Rassmann et al. 2024); models that can help assess the genome and rapidly identify disease-causing variants (De La Vega et al. 2021); and, perhaps most earth-shatteringly, models that enable accurate prediction of protein conformations (Jumper et al. 2021). As a side note, and as evidenced by the most recent Nobel Prize announcements, it is humbling to observe that some of the greatest leaps forward in the field of genetics and genomics have been made by researchers who would not describe themselves as geneticists, or even biologists, but as computer scientists and mathematicians.

In the last several years, LLMs have taken the genetic, general scientific, and the rest of the world by storm. LLM and related progress has been staggering (Flaharty et al. 2024). Recently, leaders in the field have suggested that we may be only a few years away from what some term “artificial general intelligence” and others describe as “powerful AI” (https://darioamodei.com/machines-of-loving-grace). While we can quibble over what term to use and how to best define these models (as well as have fascinating philosophical arguments about questions like consciousness, not to mention whether we will all end up like the humans of Wall-E), it is clear that these and subsequent models will profoundly impact all forms of research, including in medical genetics. In the short term, in addition to a proliferation of studies on the models themselves, it is likely that the use of these models may correlate with “research success” and impact, similar to the way in which the advent of genome sequencing ushered in a new era of discovery (Bamshad et al. 2019; Duong and Solomon 2023; Flaharty et al. 2024; McGrath et al. 2024). In fact, this may usher in a new generation of tech-savvy researchers, who may at least partially supplant those who choose not to use such tools. This type of change is an oft-repeated theme in research, but inevitably brings in controversies and questions.

In the slightly longer term (I emphasize that it may be only slightly longer due to the staggering pace of progress), the research model may flip entirely. Rather than human scientists using powerful computational tools, extremely powerful models may be the ones posing the hypotheses, developing strategies, synthesizing and analyzing data, and reporting and explaining results. Humans may be most useful for manual data collection tasks that are difficult for automated robots to perform, like certain types of field studies, or performing certain assays at the bench.

I will be honest that I am ambivalent about this. On the one hand, extremely powerful AI models could do miraculous things for human health. On the other, I admit a deep sadness that, in scientific pursuits, humans may become superfluous except for rather mundane tasks. I find it impressive but somehow disheartening to see a beautiful poem or song or painting made entirely by an AI model—perhaps this reflects a human esprit de corps (though a computer might consider it to be human bias). I feel similarly about AI models leading research.

Further, even without delving into doomsday scenarios, I worry that AI progress in biomedical research could have a number of negative effects, like exacerbating economic disparities and causing displacement due to lost jobs. Despite this, I think it is inevitable that we will soon have wide access to models that are capable of leading as well as conducting research. This may occur in a few years and be widespread in 10, or it may take a bit longer. It may occur gradually or could be propelled rapidly forward by the next big breakthrough. Ethical and regulatory questions could slow down implementation. I do not have a crystal ball and I am not a betting man. But I feel confident that this won’t be a “in my grand-kids’ lifetimes” kind of thing—it may well happen sooner than we think. Just as clinicians should think about how their practices will shift when there is routine use of accurate AI models that provide differential diagnoses, order tests, and offer management advice, we should also be prepared for how the growth of AI models will change how medical genetics research is conducted.

## 12 | Conclusions

We hope that this collection of essays provides a useful window into at least some of the ways in which experts involved in different areas of medical genetics apply and consider powerful AI technologies to the field. In conclusion, we hope to emphasize several key points.

First, the field is moving breathtakingly fast. During the months involved in assembling and editing this publication, there have been significant advances in AI, such as the availability of more powerful and versatile models, as well as new breakthroughs published in the scientific and clinical literature about applications of AI. Despite the speed, it will be imperative to understand the ramifications of AI in order to ensure that its implementation minimizes risks and maximizes benefits.

Relatedly, there is great debate occurring regarding whether it would be wise to intentionally put the reins on AI in order to better understand its effects—this holds for medicine as well as the growing number of other areas in which AI plays a role in daily life. Though the purview of this article is not meant to directly address this critical question (we will leave this to others!), many signs point to continued rapid advances while the debate rages on. The upshot is that genetics, medicine, and all of society will need to prepare for much more powerful and more “intelligent” AI systems in addition to the implementation of current systems.

Second, AI’s effects in medical genetics will not be uniform across the field. The effects may proceed at different paces and in different ways in various medical areas (e.g., for assessing radiology images in skeletal conditions vs. analyzing eye imaging for ophthalmologic conditions vs. the way AI will be deployed in other types of conditions, or the ways AI may be used in prenatal vs. pediatrics vs. adult genetics), geographic regions (e.g., United States vs. Europe vs. Asia), and in different parts of the large medical genetics ecosystem (e.g., the clinic vs. the sequencing lab vs. the research group). Each area will have to be assiduously studied and analyzed to understand and best direct AI’s uses, including to ensure that users like clinicians, researchers, and patients understand how to use (or when not to use!) AI systems. We expect that some will enjoy using AI, while others will not, which is natural and not at all a bad thing. We would argue that such choices are best made based on careful, informed consideration about the pros and cons and risks and benefits. One option that we would warn against, however, would be to try to simply ignore AI.

**FIGURE 2 |:**
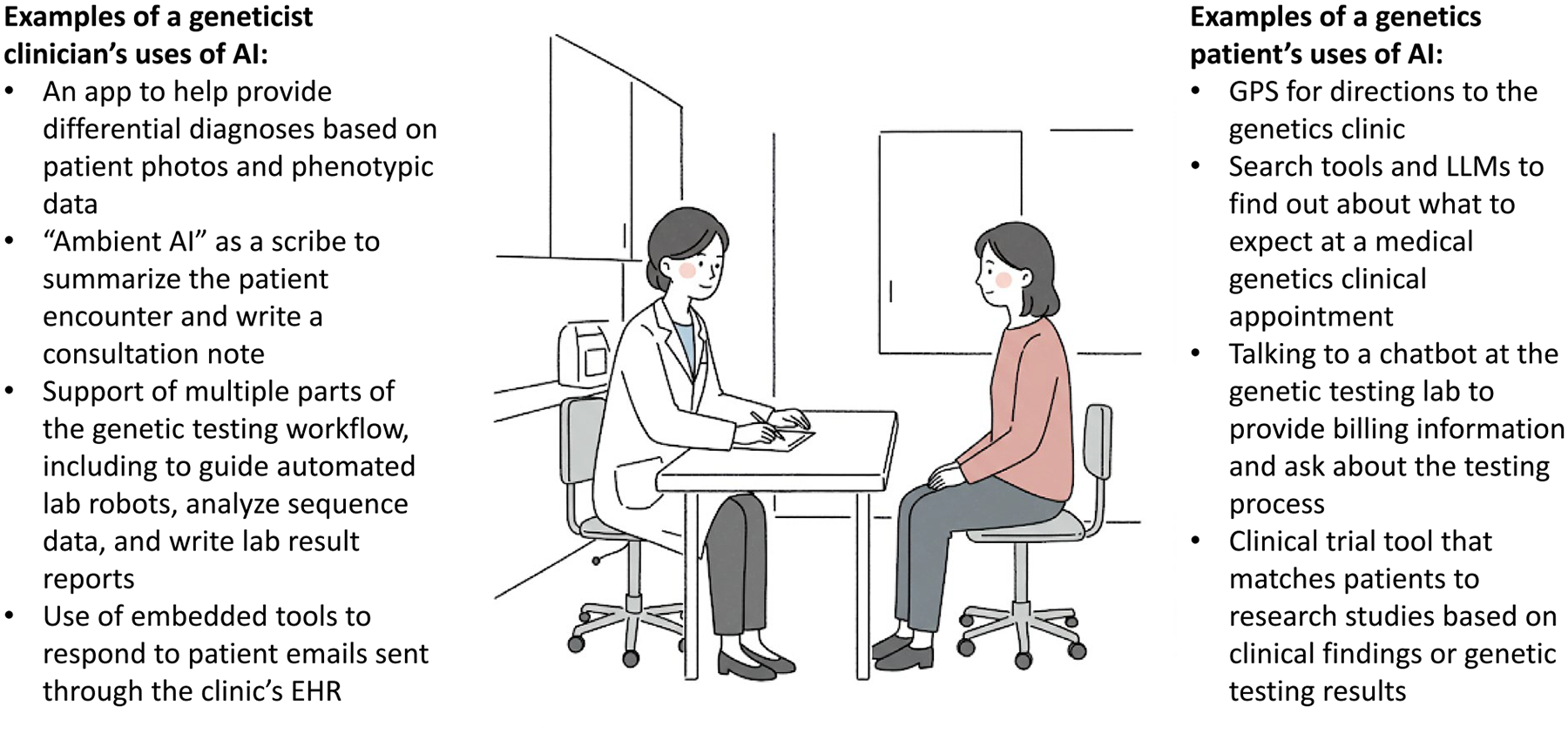
Examples of current uses of artificial intelligence (AI) in a patient/clinician encounter. Image made using Google’s Gemini model. AI, Artificial intelligence; EHR, Electronic health record; GPS, Global positioning system; LLM, Large language model.

Finally, we hope that this compendium of pieces will be of interest to many in the field of medical genetics now and going forward. We ourselves are especially interested to look back at these pieces and predictions to determine how well they may have aged. Overall, we are confident that AI will significantly affect this area of biomedicine, but exactly how this will happen will take time to materialize and parse. We hope readers may return to this collection in the years to come to determine where we may have made accurate predictions and where we may have erred! Like AI systems, we also hope to learn from our mistakes Figure 2.

## Data Availability

Data sharing not applicable to this article as no datasets were generated or analysed during the current study.
